# Incorporating Glass Wool into Urea–Formaldehyde Resin: Improvements in Fire Resistance and Dimensional Stability of Particleboards

**DOI:** 10.1021/acsomega.6c06769

**Published:** 2026-09-28

**Authors:** Michelângelo Vargas Fassarella, Izabella Luzia Silva Chaves, Emilly Soares Gomes Silva, Antônio Thiago Soares Almeida, Juarez Benigno Paes, Roberto Carlos Costa Lelis, Fabricio Gomes Gonçalves

**Affiliations:** † University of Coimbra, Department of Civil Engineering, Coimbra 3000-210, Portugal; ‡ Agrarian Sciences Department, 153595 State University of Montes Claros, Janaúba, Minas Gerais 39440-000, Brazil; § Forest and Wood Sciences Department, 680695 Federal University of Espírito Santo, Alegre, Espírito Santo 29500-000, Brazil; ∥ Institute of Forests, Products Forests Department, 67825 Federal Rural University of Rio de Janeiro, Seropédica, Rio de Janeiro 23897-000, Brazil

## Abstract

Strategies for incorporating waste materials into particleboard production represent a pursuit of sustainable, functional innovations capable of reducing environmental impacts and improving technological properties. This study investigated the incorporation of glass wool (GW) waste as a filler material in urea–formaldehyde (UF) resin and its influence on the physical and fire-retardant properties of particleboards. Panels with a nominal density of 700 kg m^–3^ were produced with different proportions of glass wool in the adhesive (T1:0%; T2:3.34%; T3:4.93%; T4:6.52%; T5:9.49%; T6:12.35%). Particleboards were produced in a hydraulic press at 160 °C with a compression force of 4.12 MPa and a pressing time of 10 min. The adhesive content was fixed at 12% in relation to the oven-dry weight of the initial raw materials (adhesive, wood particles, and glass wool). The panels were analyzed using X-ray densitometry, XRD, and FTIR. Statistical analysis included regression, analysis of variance, and the Scott-Knott test (*p* < 0.05). The results indicated that the addition of 3.39% GW to the adhesive improved the dimensional stability of the panels, while higher contents, above 9.49%, enhanced fire resistance. These findings highlight the potential of glass wool as a functional filler material in UF resin, supporting the development of safer and more sustainable particleboards.

## Graphical abstract

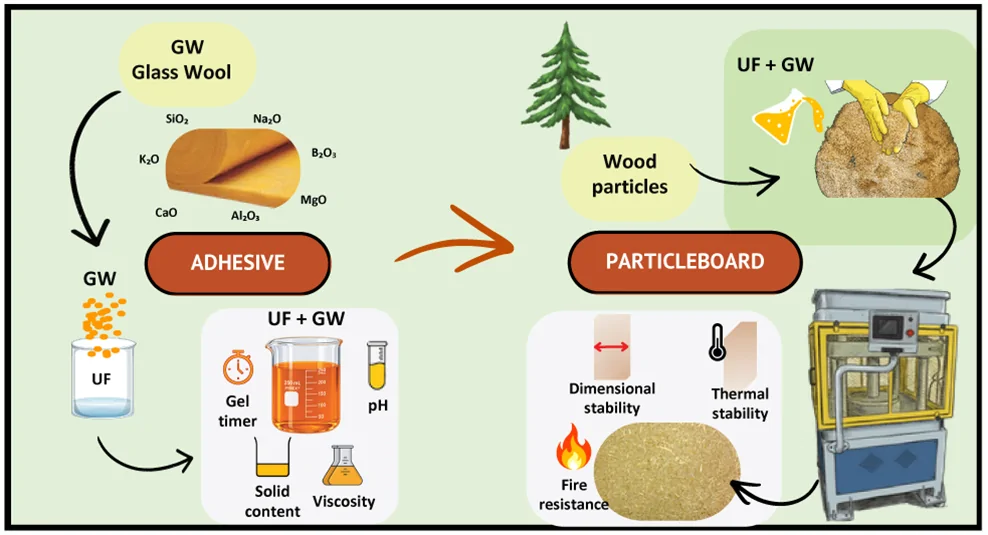

## 1 Introduction

The use of waste in the development of new products plays a significant role in industrial sustainability and the valorization of byproducts. In the reconstituted wood sector, the incorporation of waste materials as additives, extenders, and fillers in adhesive systems offers viable alternatives for technological innovation.^1–4^

Increasing attention has been directed toward the role of halogen-free fire retardants in enhancing the sustainability of building materials, particularly due to their lower environmental impact and improved recyclability compared to conventional halogenated systems.^5,6^ Similarly, ammonium polyphosphate has been used as a fire retardant in UF adhesives for particleboards.^6^ Within this framework, the present study explores the incorporation of mineral filler into the UF adhesive formulation as an alternative approach without compromising the material’s future recycling potential.

Within this broader sustainability framework, particleboards are widely used in the furniture and construction industries due to their versatility, competitive cost, and efficient use of lignocellulosic residues.^3,7,8^ However, particularly when produced with urea–formaldehyde (UF) resin, particleboards tend to exhibit lower resistance to moisture and reduced performance under high temperatures, thereby requiring strategies to improve their dimensional stability and fire resistance. The use of functional additives in UF adhesives, which is widely employed in this sector, has emerged as a promising strategy to overcome these limitations.^9–12^

Glass wool, widely used for thermal and acoustic insulation in *drywall* construction, ceilings, and piping, presents significant potential as a reusable industrial waste due to its inorganic composition, high thermal stability, and low hygroscopicity.^13–15^ Another important aspect is the search for proper postconsumer disposal, as particulate pollution, its association with other materials that hinder recycling, improper disposal, and sensitivity to contamination are key issues contributing to its environmental impact.^16,17^

Composed of silica and sodium filaments bonded with phenolic resins, glass wool is commercially available in the form of blankets, rolls, and boards.^18^ This bulky and difficult-to-reuse waste material possesses physical and chemical properties that are favorable for reintegration into polymer matrices with the potential to function as a thermal and structural barrier in composites.^17,19^

Some studies have demonstrated the potential of glass wool for applications in advanced composites, enhancing mechanical, thermal, and acoustic properties, as well as durability.^14,20–23^ Even so, with promising potential, the use of glass wool in the reconstituted wood panel sector remains underexplored.^24,25^ Consequently, its influence on the bond line integrity and multifunctional performance of these composites constitutes a significant knowledge gap. Its application as a mineral filler in synthetic adhesives for particleboards is promising, as its high specific surface area, dimensional stability, and chemical composition promote homogeneous dispersion within the adhesive matrix. Additionally, it is a friable material that allows for easy mechanical processing.

Unlike previous approaches that incorporate additives directly into panel matrices, this study innovatively incorporates glass wool waste directly into urea–formaldehyde (UF) adhesive. This approach aims to engineer interfacial interactions and thermal dynamics at the bondline. The research involved the characterization of glass wool residues and the evaluation of their effects on physical and hygroscopic properties and flame behavior, contributing to the advancement of sustainable solutions in the reconstituted panel industry.

## 2 Materials and Methods

### 2.1 Raw Material Acquisition and Particleboard Production

Glass wool waste was obtained from discarded refrigeration equipment collected in southern Espírito Santo, Brazil. The material was manually cleaned to remove contaminants and mechanically reduced to fragments of approximately 10 × 10 mm to facilitate its incorporation into the adhesive system.

The wood was obtained in the form of commercial boards, cut perpendicular to the grain (≈20 × 9 × 2 cm), saturated in water, and subsequently processed into flakes (≈90 × 20 × 1.7 mm) using a particle generator (MARCONI, MA 685, Piracicaba, Brazil). The particles were air-dried, processed in a hammer mill (TIGRE, A4, São Paulo, Brazil), and classified using a vibrating sieve (SOLOCAP, M12, Belo Horizonte, Brazil), retaining only material between 2.0 and 4.0 mm. After selection, the particles were oven-dried (80 ± 2 °C) to 7% moisture content and stored in airtight bags.

The urea–formaldehyde (UF) adhesive and ammonium sulfate catalyst were purchased from a national supplier. The addition of glass wool as a filler material in the UF adhesive followed the proportions established for each treatment (Table 1). The application of glass wool to the UF adhesive was manually homogenized inside a plastic basin, using gloves.^8,26^

**Table 1:** Experimental Design Based on the Percentage of Glass Wool Filling in the Urea–Formaldehyde Adhesive, with Respective Mass and Characteristics of the Adhesive Used after the Addition of Wool^a^

			Mass of matetrial (g)			Adhesive property^27^			
Treatments	Glass wool content in the particleboard (%)	Glass wool content in the adhesive (%)	Glass wool	Wood particle	Adhesive	pH	SC	Visc.	GT
T1	0	0	0	1274.57	152.95	7.91	62.88	1141.98	83.2
T2	0.50	3.34	8.37	1267.82	152.14	8.20	67.41	1288.88	68.2
T3	0.75	4.93	12.52	1264.48	151.74	8.15	63.79	1671.96	93.8
T4	1.00	6.52	16.77	1261.05	151.33	8.29	68.14	1747.26	67.2
T5	1.50	9.49	25.09	1254,34	150.02	8.37	68.63	1960.16	78.4
T6	2.00	12.35	33.56	1247.51	149.70	8.59	71.85	7970.72	111.2

a pH: hydrogenionic potential; SC: solid content (%); Visc.: viscosity (cP); and GT: gel time (s).

The panels were produced using a hot hydraulic press at a pressure of 4.12 MPa, a temperature of 160 °C, and a pressing time of 10 min (press closing time: 55 s). The panels had an area of 0.180625 m^2^ (nominal dimensions of 42.5 cm × 42.5 cm. Metal separators were used to limit the thickness of the panels to 1.2 cm. The target density was 700 kg m^–3^. Three replicates were produced for each treatment, totaling 18 panels. For all treatments, a fixed resin content of 12% (dry basis) was used. After hygroscopic stabilization in a climate-controlled room (65 ± 5% relative humidity and 25 ± 3 °C) until equilibrium was reached (minimum 72 h), test specimens were prepared according to the specific standards required for each test (Table 2).

**Table 2:** Specimen Dimensions, Number of Specimens per Treatment, and Standards Associated with Each Test

Test	Specimen dimensions (mm)	Number of specimens per treatment	Standards
Density (ρRx)	50 × 50	12	NBR 14810–2 ^28^
Water absorption (WA)	50 × 50	11	
Thickness swelling (TS)	50 × 50	9	
Flammability (Flam.)	310 × 50	15	BS EN ISO 11925–2 ^29^

### 2.2 Physical Properties of the Filler Material and Particleboards

#### 2.2.1 X-ray Diffraction Analysis

To characterize the glass wool residue and the particleboards, X-ray diffraction (XRD) analyses were performed using a diffractometer (RIGAKU Miniflex 600, Osaka, Japan) with Cu Kα radiation (λ = 1.54 Å). Powdered samples were scanned over a 2θ range of 5° to 70°, with a scan rate of 2° min^–1^ and a step size of 0.02°.

#### 2.2.2 Density (ρRx)

The X-ray densitometry test was performed using a densitometer (GRECON, DAX6000, Germany), allowing for the determination of apparent density profiles of the particleboards, as well as the calculation of mean, maximum, and minimum densities.^30^

Based on the data obtained from the X-ray densitometry analysis, equations were fitted to estimate the influence of maximum and minimum densities on the mean density of the panels for each treatment. General equations were calculated as weighted averages of the linear and angular coefficients fitted to each treatment.

#### 2.2.3 Water Absorption (WA) and Thickness Swelling (TS)

The thickness was measured with a micrometer at the center and at four distinct edge positions of each specimen. The mass was determined using an analytical balance. After 24 h of immersion in distilled water, and with excess water removed using absorbent paper, thickness, mass, and edge measurements were repeated.

TS was calculated based on the difference between the final and initial thicknesses, while WA was determined by comparing the final and initial masses. The procedures followed standardized methods for the 24-h water immersion test, and both results were expressed as percentages.

### 2.3 Thermogravimetric Analysis

The thermal degradation behavior of glass wool and particleboards was evaluated through thermogravimetric analysis (TGA) and derivative thermogravimetry (DTG) of powdered samples. The analysis was carried out using a thermogravimetric analyzer (SETARAM, LabSys Evo, Caluire-et-Cuire, France) under heating conditions from room temperature to 800 °C at a heating rate of 10 °C min^–1^ in a nitrogen atmosphere.

### 2.4 Flammability Test of Particleboards

To assess the flammability of the material, a total of 90 specimens previously used in static bending tests (310 mm long) that did not exceed the limit of proportionality were used. The pressure burner was replaced with a Bunsen burner made from the specifications of BS EN ISO 11925-2 standard,^29^ with a total of 15 repetitions per treatment.

The samples were suspended vertically for the test, with the lower part of each specimen directly exposed to a flame 18 ± 2 mm in height generated by a Bunsen burner (Figure 1A). The specimens were positioned 5 mm away from the heat source, ensuring that the flame uniformly reached both the centerline and edges. To standardize exposure, the heat source was placed 4 cm above the bottom edge of each specimen. The specimen was adjusted at a 45° inclination in relation to the Bunsen burner, which was also properly adjusted to produce a stable and visible flame.

**Figure 1:**
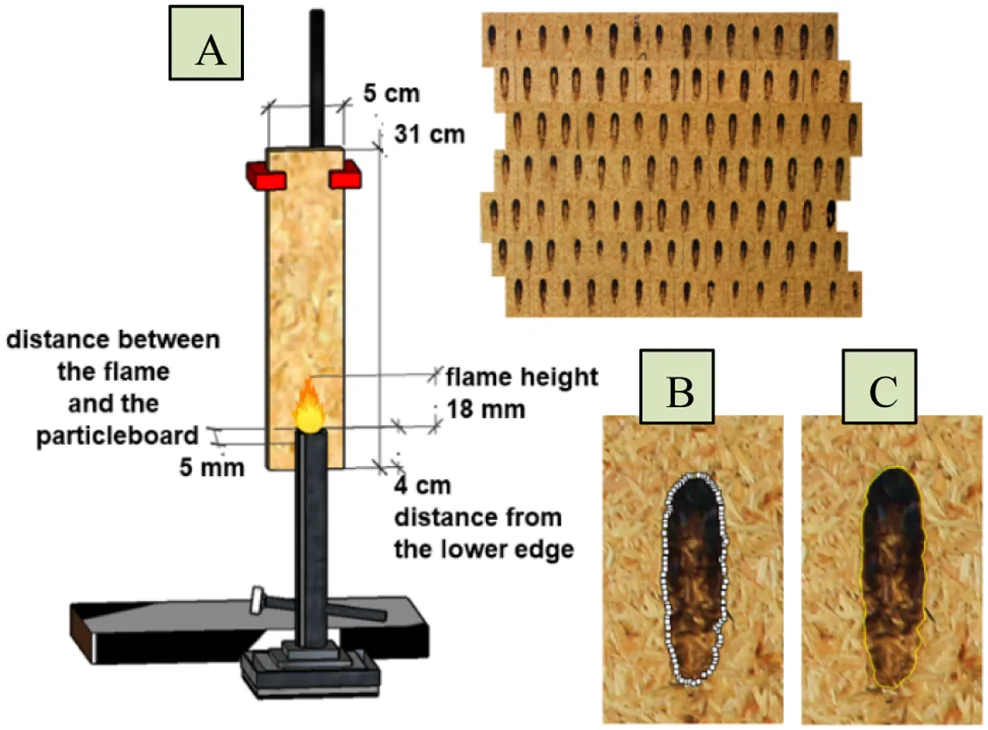
Flammability test. A: Specimen exposed to the flame generated by the Bunsen burner; B: subsets of points used to define the polygon of the charred area after 30 s of flame exposure; C: measurement of the charred area using ImageJ *software*. Source: Modified fromFassarella.^31^

After 30 s of flame exposure, the charred area (mm^2^) on each specimen was measured using ImageJ software. This program enabled precise area measurement by defining a polygon on the captured image (Figure 1B,C). The measurements provided quantitative data on the extent of damage caused by the flame and allowed for a detailed analysis of the flammability of the particleboards.

### 2.5 Chemical Characterization of the Particleboards

Samples of the glass wool residue and the produced particleboards were characterized by Fourier transform infrared spectroscopy (FTIR), following the specific methodology described by Delatorre et al.^32^ The spectra were obtained using a spectrophotometer (BRUKER, Tensor 27, Germany), within the mid-infrared frequency range (4000–400 cm^–1^), and processed between 4000 and 600 cm^–1^ with a resolution of 4 cm^–1^ using attenuated total reflectance (ATR).

### 2.6 Statistical Analysis

Statistical analyses were conducted individually for each test, with outlier removal based on the interquartile range (IQR) calculated from the first and third quartiles using Excel. Values outside the minimum and maximum thresholds were considered *outliers* and were excluded.

The analyses followed a completely randomized design. Initially, regression analysis was performed within the variance framework; when no statistical significance was observed, the procedure continued with the analysis of variance followed by the Scott-Knott test.

## 3 Results and Discussion

### 3.1 Thermogravimetric Analysis

As observed in the thermogravimetric (TG) curve of the glass wool sample (Figure 2), two mass-loss regions and a small apparent mass increase were identified. The first occurred between 52 and 242 °C, with a 1.7% apparent increase in sample mass. Up to approximately 200 °C, the derivative thermogravimetric (DTG) curve revealed a low-temperature feature associated with the rate of mass variation.

**Figure 2:**
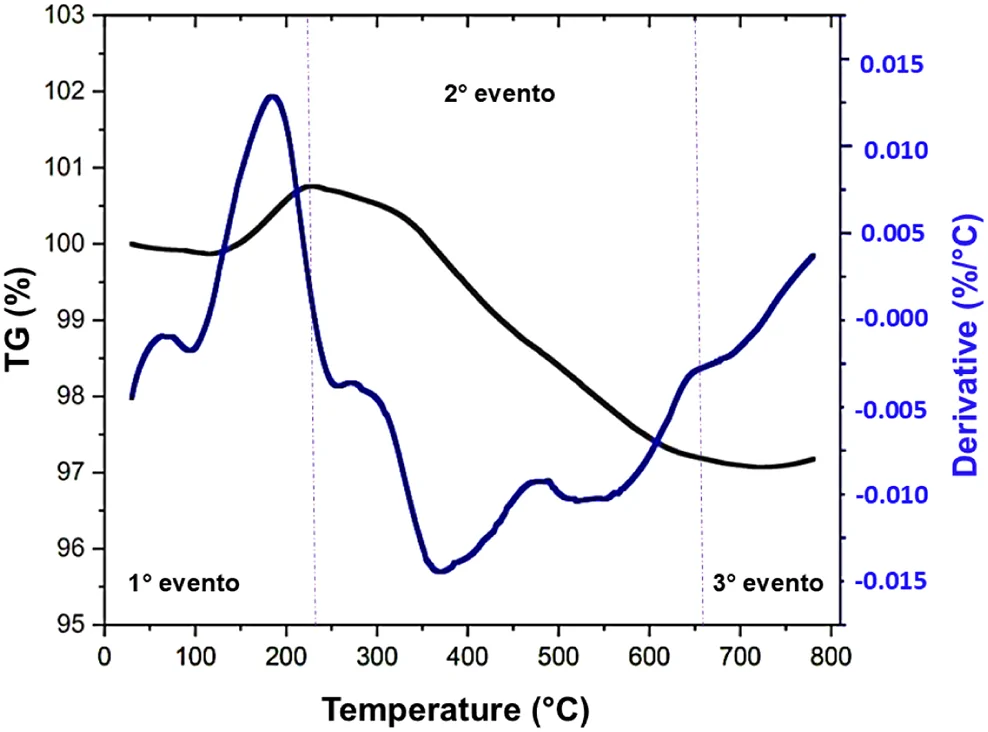
TG/DTG curve of glass wool residue.

The slight mass gain identified in the TG curve (first event) can be attributed to the adsorption of gases or volatile compounds resulting from oxidation and surface chemical reactions in the sample.^33^ In the second event of the TG curve, above 242 °C, there is a gradual mass reduction extending up to 654 °C, likely associated with the decomposition of binders and other components.^34,35^ Beyond this temperature range, the sample exhibited thermally stable behavior, with no significant mass variation up to 800 °C. The mass loss of less than 3% confirms the high thermal stability of the glass wool residue.

In contrast, particleboards exhibited multistage degradation behavior (Figure 3A and B, respectively). The first event, at temperatures below 120 °C, is associated with mass loss due to the evaporation of moisture and low thermal stability volatile compounds present in the wood samples.^36^ Between approximately 120 and 230 °C, the particleboards showed relatively limited mass variation, followed by the main degradation stage from approximately 230 to 360 °C..

**Figure 3:**
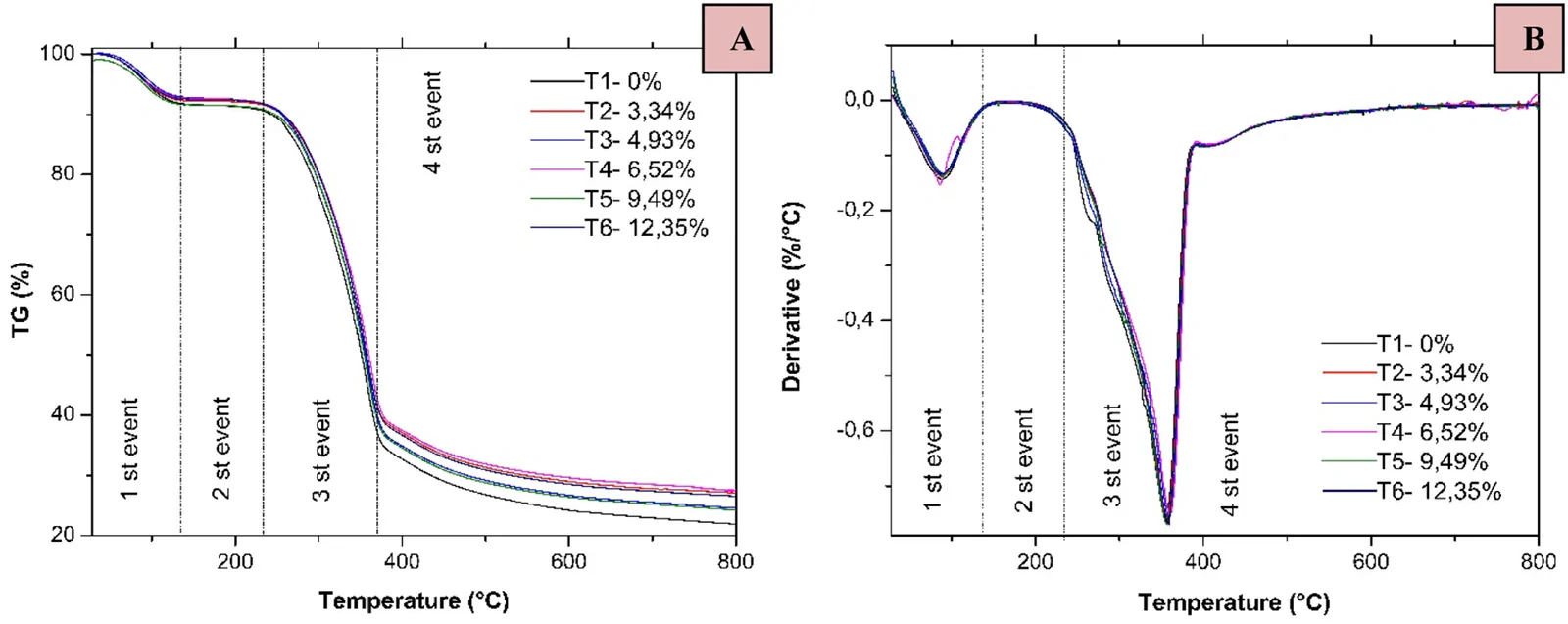
TG (A) and DTG (B) curves of the particleboards.

The main degradation stage occurred between approximately 230 and 360 °C, characterized by a significant mass loss and a prominent peak in the DTG curve, indicating the maximum rate of mass loss.^37^ Within this temperature range, degradation of hemicelluloses occurs first, followed by cellulose decomposition and the breakdown of polymer chains in the adhesive matrix, resulting in the formation of carbonaceous residues.^38^

Above 360 °C, a deceleration in the degradation rate was observed across all treatments. However, panels produced without mineral filler exhibited greater residual mass loss. The higher final residue observed in the glass-wool-containing panels is consistent with the contribution of a thermally stable inorganic fraction introduced with the residue. Therefore, residue values alone were not interpreted as evidence of improved thermal stability. To distinguish the contribution of the inorganic filler from changes in the degradation behavior of the organic fraction, T5%, Tonset, Tmax, DTG, and residue at 800 °C were compared among the treatments (Table 3).

**Table 3:** Thermogravimetric Parameters and Residue Content of the Composites as a Function of Glass Wool Content

Treatment	Glass wool (%)	T5% mass loss (°C)	Tonset (°C)	Tmax (°C)	DTG	Residue at 800 °C (%)
T1	0	94.85	178.3	356.5	–0.768	21.83
T2	0.50	96.35	201.3	356.8	–0.758	27.05
T3	0.75	101.6	172.6	359.2	–0.755	24.48
T4	1.00	94.66	187.2	359.8	–0.753	27.68
T5	1.50	91.10	189.5	356.5	–0.771	24.21
T6	2.00	97.12	188.7	359.3	–0.770	26.52

The Tmax values remained relatively close among the treatments, ranging from 356.5 to 359.8 °C, indicating that the incorporation of glass wool did not substantially shift the temperature of maximum degradation. Likewise, neither T5% nor Tonset showed a systematic trend with increasing glass wool content. Although the glass-wool-containing panels exhibited higher residues at 800 °C than T1, this increase should not be interpreted directly as enhanced thermal stability, since it is partly attributable to the thermally stable inorganic fraction of the filler. Therefore, the TG/DTG parameters suggest that the addition of glass wool did not substantially modify the main degradation temperature of the organic fraction under the conditions investigated.

Similar results regarding thermal stability were observed by Torun et al.^39^ and^10^ when evaluating lignocellulosic composites with the addition of mineral fillers in urea–formaldehyde and melamine–formaldehyde adhesive matrices. These studies show that mineral fillers can alter residue yield and, in some cases, thermal degradation behavior; however, the contribution of the inorganic fraction must be separated from changes in the organic fraction before an improved thermal stability is inferred.

### 3.2 X-ray Diffraction Analysis

The X-ray diffraction (XRD) profile analysis (Figure 4) confirms cellulose crystallinity peaks in the particleboard sample. In contrast, the glass wool residue sample exhibited an amorphous halo with a lack of sharp, well-defined peaks, reflecting the material’s noncrystalline nature.^13,15,24,40^

**Figure 4:**
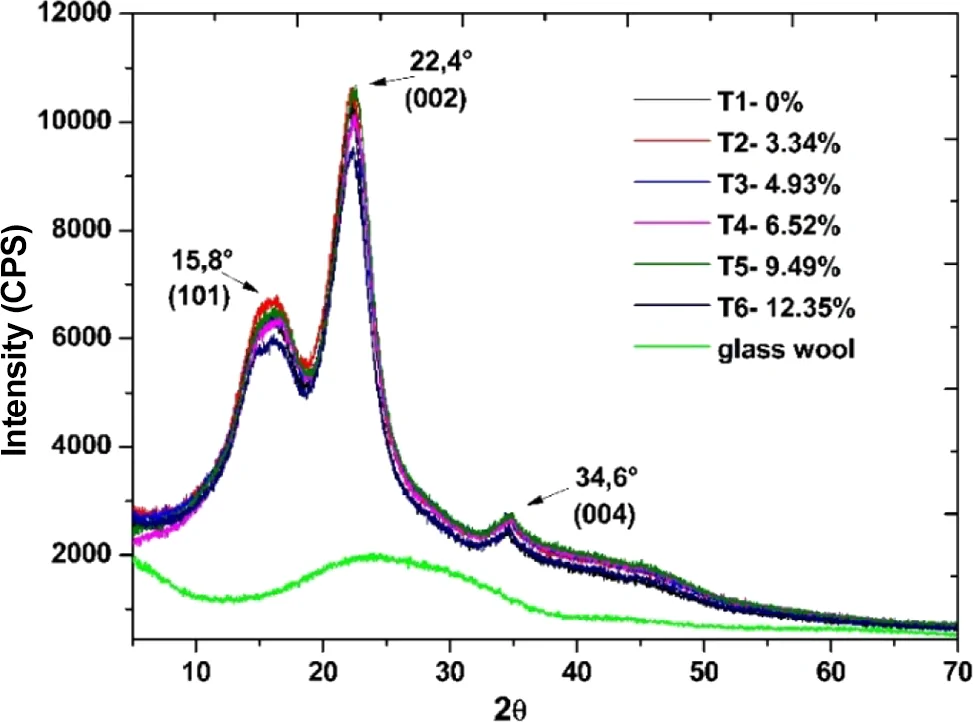
X-ray diffraction patterns of particleboards and glass wool residue.

The X-ray diffraction of the particleboards revealed peaks at 2θ values of 15.8° and 22.4°, attributed to semicrystalline structures, consistent with the (101) and (002) planes of cellulose I.^41^ The presence of H^+^ ions during acid curing promotes the extension of linear oligomers by condensation and favors the formation of crystalline domains at the interface of the adhesion systems.^42^ However, the present qualitative XRD data do not allow the formation of new crystalline domains at the adhesive interface to be inferred.

The incorporation of glass wool into the adhesive did not significantly alter the crystalline structure of the particleboards. Such structural characteristics can contribute to the enhancement of the mechanical and thermal properties of the composites.^43^

An additional peak at approximately 2θ ≈ 34.6° was detected in all panels. This peak is consistent with the (004) plane of cellulose I and is therefore more likely associated with the lignocellulosic fraction than with crystalline domains of the UF resin. According to Zuber et al.,^44^ the broad diffraction feature observed at approximately 32°–37° is predominantly associated with an amorphous component rather than well-ordered crystalline domains. The similar diffraction profiles among the panels indicate that glass wool incorporation did not substantially alter the crystalline–amorphous character of the lignocellulosic fraction within the resolution of the qualitative XRD analysis.

### 3.3 Physical Properties of the Particleboards

#### 3.3.1 Density

The analysis of variance for the apparent density of the particleboards, considering the different glass wool contents, revealed no statistically significant differences (*p* > 0.05), a result supported by the Scott–Knott grouping test. With a nominal density of 700 kg m^–3^, values ranging from 712 to 732 kg m^–3^ represent an increase of up to 4.6% compared to the target parameter (Table 4).

**Table 4:** Average Apparent Density Values of the Produced Particleboards for Different Proportions of Glass Wool Added to the Urea–Formaldehyde Adhesive

Treatment^a^	Density (kg m^–3^)	Standard deviation	F-test statistic
T1 – 0%	726.3	41.58	0.5282^ns^
T2 – 3.34%	730.0	18.85	
T3 – 4.93%	718.3	28.65	
T4 – 6.52%	712.9	11.19	
T5 – 9.49%	725.2	19.97	
T6 – 12.35%	732.1	42.01	
**Mean**	**724.1**	**29.10**	

a Treatment: percentage of glass wool in the urea–formaldehyde adhesive. ^ns^: not significant (*p* > 0.05).

Based on data obtained from the X-ray densitometry analysis, the fitted equations for maximum and minimum densities showed slope values close to 1, indicating a stable linear relationship across treatments (Table 5).

**Table 5:** Equation Estimated for the Maximum and Minimum Apparent Density from the Average Density of Particleboards

Treatment^a^	Maximum density estimated equation	R^2^	Minimum density estimated equation	R^2^
T1 – 0%	Ŷ = 16.033 + 0.8600*X_max_	0.97	Ŷ = 34.632 + 1.0575*X_min_	0.95
T2 – 3.34%	Ŷ = 49.559 + 1.0737*X_max_	0.95	Ŷ = −47.680 + 0.9636*X_min_	0.96
T3 – 4.93%	Ŷ = 238.78 + 0.8224*X_max_	0.85	Ŷ = −31.360 + 0.9289*X_min_	0.95
T4 – 6.52%	Ŷ = 130.49 + 0.9621*X_max_	0.94	Ŷ = −45.492 + 0.9547*X_min_	0.98
T5 – 9.49%	Ŷ = 50.722 + 1.0818*X_max_	0.80	Ŷ = −51.238 + 0.9586*X_min_	0.93
T6 – 12.35%	Ŷ = 14.908 + 1.1100*X_max_	0.92	Ŷ = −25.370 + 0.9287*X_min_	0.97
**General**	Ŷ = 95.780 + 1.01102^a^X_**max**_	**0.87**	Ŷ = −41.612 + 0.9503^a^X_**min**_	**0.94**

a Treatment: percentage of glass wool in the urea–formaldehyde adhesive. R^2^: Coefficient of determination indicating the proportion of variability in the dependent variable explained by the model. Ŷ: Estimated mean density (kg m^–3^); *X*_max_: Maximum mean density; *X*_min_: Minimum mean density; *: significant (*p* < 0.05).

The results indicate that, regardless of the glass wool content in the adhesives—which differentiates the treatments—the overall density behavior throughout the thickness of the panels followed the expected pattern.

A practical analysis of these models allows for the evaluation of structural quality parameters of the panels without the need for individual measurements for each treatment. Furthermore, the high linearity observed reinforces the reliability of X-ray densitometry as a nondestructive testing technique for the characterization of composite materials.^30^

The significant difference between the linear coefficients of the maximum and minimum densities highlights the heterogeneity in the density distribution, which is consistent with findings from similar studies on reconstituted wood panels.^45,46^

The density profile describes the variation in panel density along its thickness.^30^ In reconstituted panels, particularly during hot pressing, the outer layers experience greater compression, resulting in higher surface densification on both faces of the panel, while the core layer exhibits lower density.^38,47,48^ This leads to an “M-shaped” density profile across the specimen’s thickness (Figure 5).

**Figure 5:**
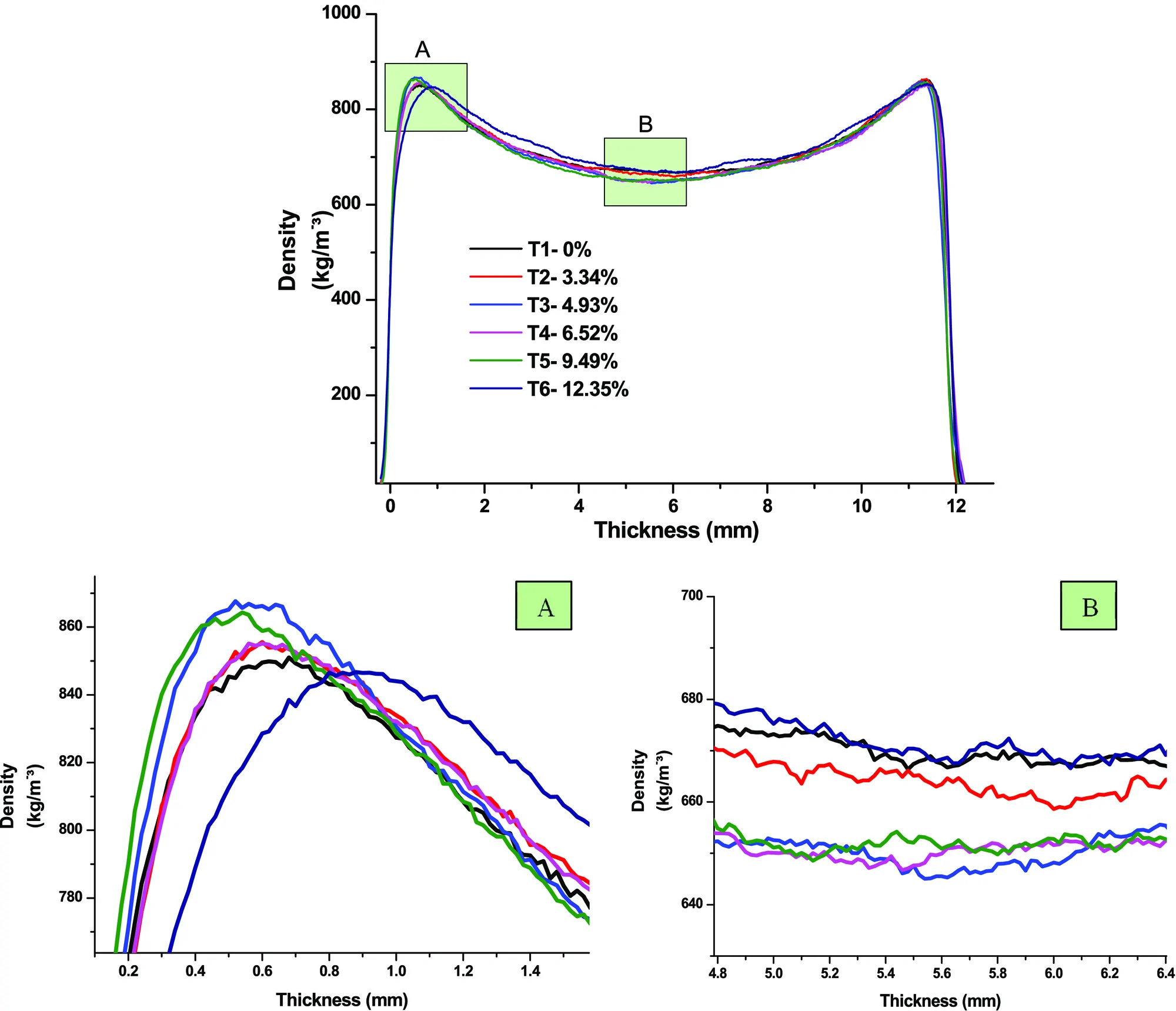
Average apparent density profiles along the thickness of the particleboards as a function of the glass wool content added to the urea–formaldehyde adhesive. A: Upper surface of the panel relative to the plane of the electric hydraulic press; B: Central region of the panel.

Density influences the mechanical properties, adhesive efficiency, and dimensional stability of particleboards.^46,49^ Moreover, the surface layer density in reconstituted wood panels plays a key role in fire resistance.^4,9^

The uniformity observed in the slopes of the fitted equations, along with the behavior of the apparent density profile, confirms effective control over the particleboard manufacturing process, ensuring consistent structural performance. These results highlight the importance of density control as a critical parameter in panel production, as it is directly linked to product quality and final performance.^45,50^ Figure 5A and B represents a zoom of the density profile to better visualize the behavior of the average profile between the different treatments.

The maintenance of density profiles regardless of the filler content suggests that the incorporation of glass wool did not interfere with mat consolidation during pressing, which is important for the quality of the panel.

#### 3.3.2 Water Absorption (WA) and Thickness Swelling (TS)

In the water absorption and thickness swelling tests after 24 h of immersion, linear regression analysis showed that the addition of glass wool to the UF adhesive significantly affected the physical properties of the particleboards (*p* < 0.05). Both WA and TS increased linearly with the proportion of incorporated glass wool (Figure 6A and B), with R^2^aj. values of 0.878 and 0.718, respectively.

**Figure 6:**
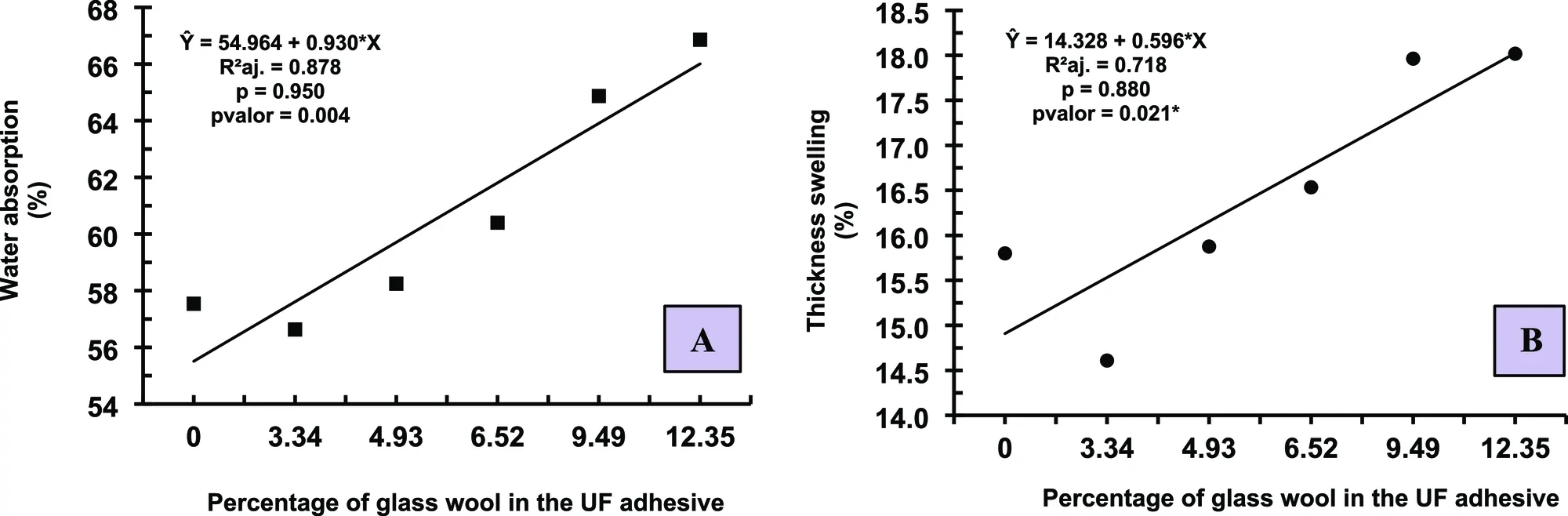
Relationship between water absorption (A) and thickness swelling (B) of particleboards as a function of the percentage of glass wool added to the urea–formaldehyde (UF) adhesive. **: significant at 1% probability; *: significant (*p* < 0.05); Ŷ: estimated water absorption or thickness swelling (%); X: percentage of glass wool added to the urea–formaldehyde adhesive (%); R^2^aj.: adjusted coefficient of determination; *p value*: probability associated with the test.

Particleboards with higher percentages of glass wool residue incorporated into the UF adhesive showed a greater water absorption and thickness swelling. The increase in the residue content affected the adhesive interaction and structural morphology of the panel, suggesting variations in dispersion and contact between the adhesive and wood particles, which promoted the formation of hygroscopic bonds.

Water absorption ranged from 56.6% to 66.8%, with emphasis on the addition above 6.52% glass wool content, supporting the hypothesis of an increased porosity. It is important to note that the high extractive content from the *Pinus* spp. particles affects the hygroscopicity and permeability of the panel, as high concentrations of these compounds impair particle bonding, leading to low adhesive strength.^51,52^

Finer wood particles tend to reduce the internal porosity of particleboards, contributing to a better hydrophobic performance by minimizing the surface area available for water absorption. However, a larger surface area also requires a greater amount of adhesive to ensure complete coverage and effective bonding between particles.^53,54^

In this context, considering the low slenderness ratio of the particles used in panel production, it is plausible to suggest that treatments with higher filler content—and consequently lower UF adhesive proportions—may have hindered uniform adhesive distribution among the particles, compromising moisture resistance.

The thickness swelling (TS) results followed a trend similar to that of water absorption, with values ranging from 14.61% to 18.02%. A smaller addition of glass wool tends to the UF adhesive to improved physical properties of the particleboards (Figure 6B).

Treatments T5 and T6 exhibited the highest thickness swelling (TS) values −17.65% and 18.02%, respectively. Nevertheless, these values remained close to the threshold established by the Brazilian standard NBR 14810-2,^28^ which sets the limit for thickness swelling after 24 h of water immersion at 18%.

According to Bazzetto et al.,^53^ thickness swelling in particleboards can be attributed to the release of compressive stresses, which tend to increase with higher compaction rates. On the other hand, greater amounts of adhesive in panels reduce thickness swelling values.^55,56^ Therefore, a decrease in the glue line coverage can lead to areas of higher water absorption and lower dimensional stability in the panel.

The good performance of the evaluated panels, when compared with data from the literature (Table 6), which compiles studies on dimensional stability properties (water absorption and thickness swelling), reinforces the importance of paraffin, the influence of raw materials, processing conditions, and production parameters.

**Table 6:** Literature Review of Water Absorption (AA) and Thickness Swelling (IE) of Particleboards Produced with Different Wood Species and Adhesives

			**Property**		
Panel	Adhesive	Wood species	WA^a^ (%)	TS (%)	Source
Agl	UF + pe	*Pinus taeda* (bark)	78.24	20.65	Luckman et al.^57^
		*Eucalyptus grandis* x *Eucalyptus urophylla*	115.80	38.70	Souza et al.^58^
	UF	*Pinus oocarpa*	≈77.0	≈11.0	Narciso et al.^59^
			104.03	21.3	Furtini et al.^52^
	UF + pe	*Pinus patula*	18.51	26.75	Rios et al.^60^
		*Pinus taeda*	29.66	9.96	Iwakiri et al.^61^
	UF	*Pinus oocarpa*	65.05	22.46	Brito et al.^62^
Agl^3^	UF	*Pinus sylvestris*	≈82.0	≈29.0	Wronka and Kowaluk^48^
	UF + pe		83.2	19.9	Benthien et al.^63^
Agl	UF	*Pinus* sp. *+ choupo*	100.0	54.0	Karagiannidis et al.^64^
		*Pinus pinaster*	91.54	13.4	Benhamou et al.^65^
		*Pinus* sp. + Choupo	-	21.4	Onat and Kelleci^66^
		*Gmelina arborea* Roxb.	96.79	28.38	Nuryawan et al.^67^
		*Hevea brasiliensis*	46.07	28.01	Ghani et al.^68^
			89.47	29.36	Sulaiman et al.^69^
	UF + NH_4_Cl	*Hevea brasiliensis*	46.74	28.40	Ghani et al.^70^

a WA: water absorption (24 h); TS: thickness swelling (24 h); Agl: particleboard; Agl^3^: three-layer particleboard; UF: urea–formaldehyde; pe: paraffin emulsion; -: not found; ≈: approximate value.

It is worth noting that the UF adhesive, due to its high hygroscopicity, requires the incorporation of paraffin to improve its physical properties related to water absorption and thickness swelling.^71^ Therefore, it is crucial to balance the adhesive formulation with the characteristics of the material to ensure an effective bond and enhance the physical properties of the panels.

Overall, the decrease in the percentage of urea–formaldehyde due to the increased glass wool filler content may have compromised the effective wetting of the wood particles. Given the high specific surface area of the filler material, the adhesive demand increases, which leads to reduced wetting of the wood particles.^72,73^

### 3.4 Particleboard Flammability

The mean results of the treatments evaluated in the flammability test are presented in Figure 7. Analysis of variance (ANOVA) revealed significant differences among the treatments (F, *p* < 0.01). When grouped using the Scott-Knott test (*p* < 0.05), the treatments were separated into two statistically distinct groups, confirming significant differences in performance.

**Figure 7:**
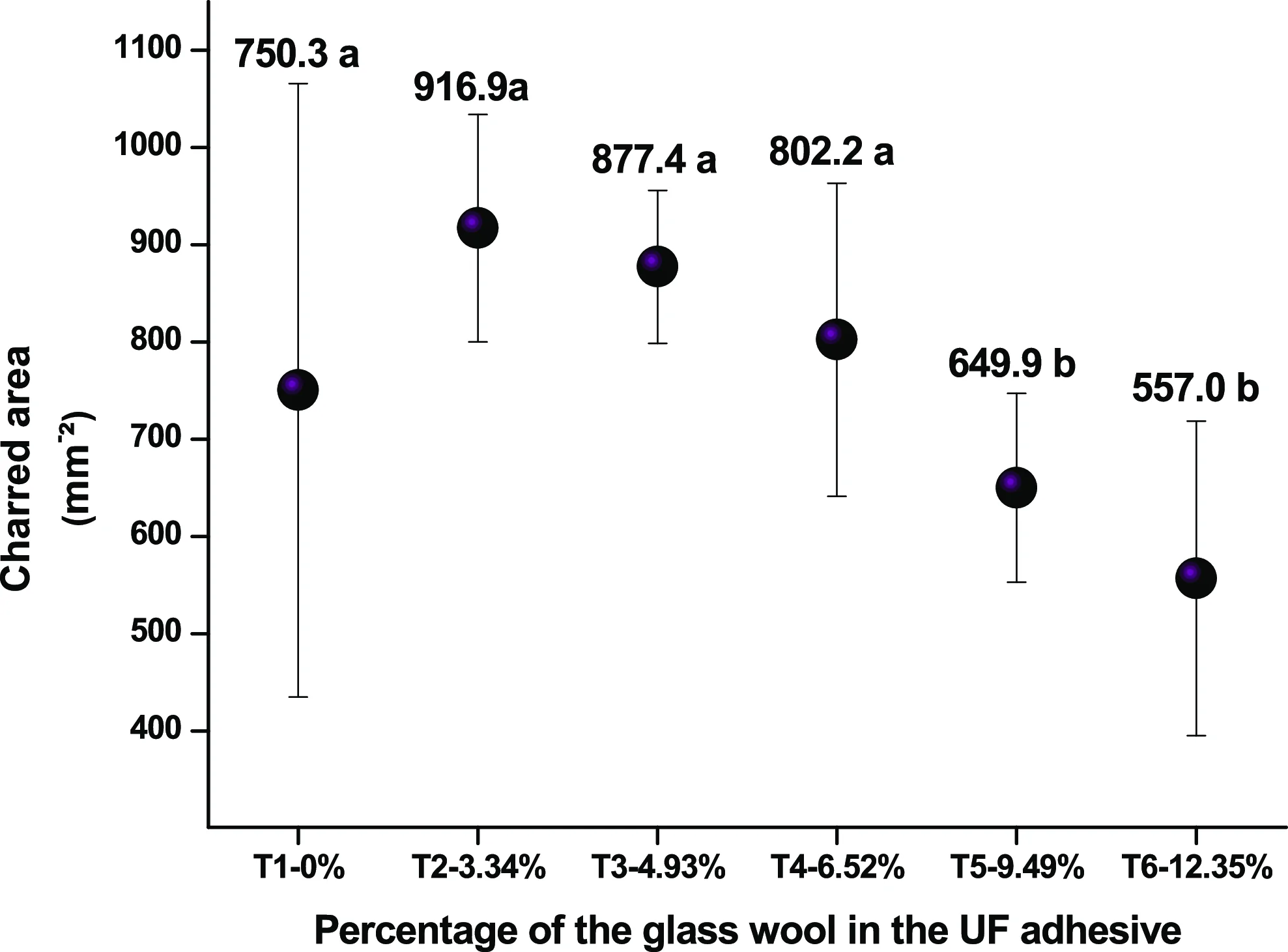
Mean values of the flammability test for particleboards, showing the charred area (mm^2^) as a function of the percentage of glass wool added to the urea–formaldehyde (UF) adhesive. Means followed by the same letter do not differ significantly (Scott–Knott, *p* > 0.05). Bars correspond to standard deviation.

Treatments T5 and T6, containing 9.49% and 12.35% glass wool in the adhesive, showed the smallest charred areas −649.9 mm^2^ and 557.0 mm^2^, respectively—significantly outperforming the other treatments. Although BS EN ISO 11925-2 primarily assesses ignitability, the charred area was adopted in this study as a comparative parameter to estimate the extent of flame propagation and surface degradation among the treatments. This minimum threshold of filler content, sufficient to improve the flame spread behavior of the particleboards, may be attributed to the more uniform distribution of glass wool fibers throughout the panel matrix, limiting flame propagation across the panel surface.

This behavior is attributed to the inorganic and noncombustible nature of glass wool, which acts as a thermal barrier and reduces heat transfer to the underlying material. The high silica content in glass wool residues—an inorganic and noncombustible material—contributed to the formation of a barrier that reduced fire interaction with the surface of the particleboards. Additionally, the distribution of fibers within the matrix may contribute to limiting the continuity of the combustible pathways.

The results of this study align with recent investigations aimed at improving the fire resistance of particleboards, whether by using alternative binders^4,10,35,74^ or by employing different production methods.^75^

The fire performance assessment was based on surface flame exposure and charred area analysis, providing an initial indication of fire resistance and enabling comparison among treatments (comparative reduction in surface flame damage). However, a comprehensive evaluation requires standardized methods such as the limiting oxygen index (LOI), cone calorimetry, and smoke density analysis. The incorporation of such techniques in future studies would enable a more detailed understanding of the combustion mechanisms of the developed composites.

### 3.5 Chemical Characterization

The composition of the glass wool residue, analyzed by Fourier-transform infrared spectroscopy (FTIR) (Figure 8), revealed an absorption band at around 947 cm^–1^, attributed to the stretching vibrations of Si–O^–^ in silanol groups (Si–OH), which are characteristic of materials composed of amorphous silica networks.^76^ The band identified at 765 cm^–1^ corresponds to bending vibrations of Si–O–Si bonds, which are commonly present in the structural network of glass materials.^77,78^

**Figure 8:**
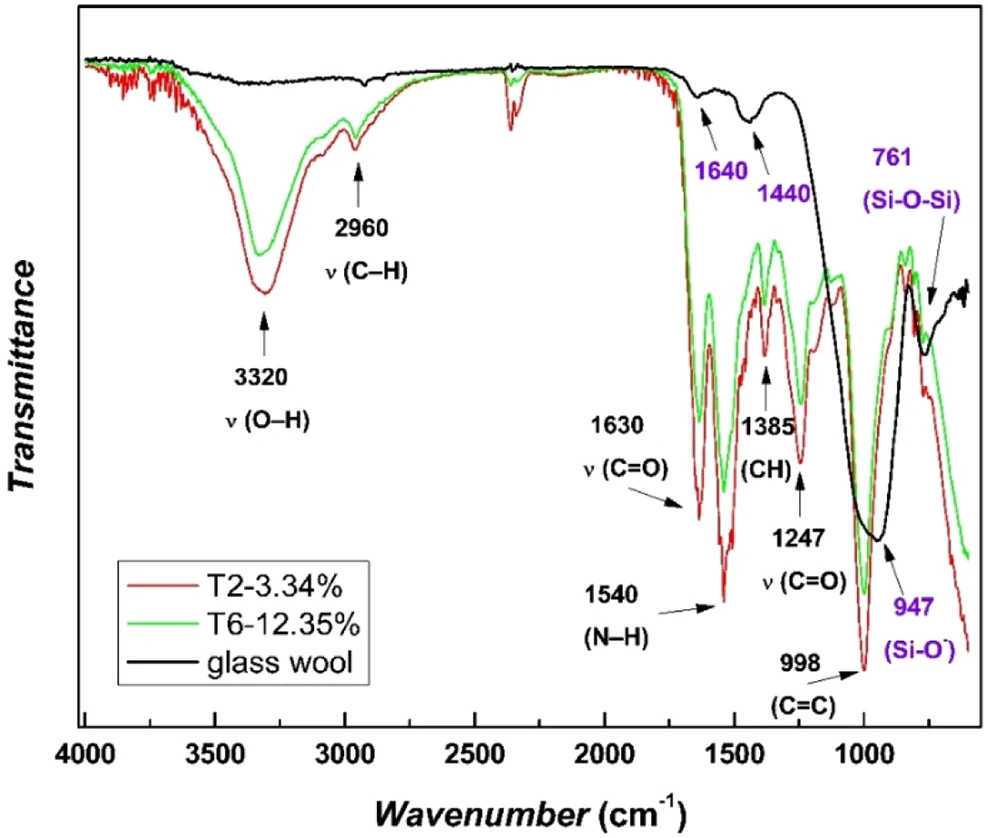
Normalized FTIR spectra of particleboards containing 3.34% (T2) and 12.35% (T6) glass wool and of the glass wool residue, highlighting the main absorption bands.

The comparison between T2 and T6 adhesives is particularly relevant because the glass wool content increases from 3.34% to 12.35%. The spectrum of T6 shows a more evident contribution from the inorganic phase in the fingerprint region than T2, particularly around the Si–O-related region. However, the spectral features remain strongly overlapped with bands arising from the lignocellulosic and UF components, preventing a definitive assignment of the observed intensity variations to specific interfacial chemical reactions.

The main FTIR bands identified for all particleboard treatments are summarized in Figure 9. Overall, the spectra exhibited similar band positions across the treatments, with no distinct new absorption bands emerging with increasing glass wool content.

**Figure 9:**
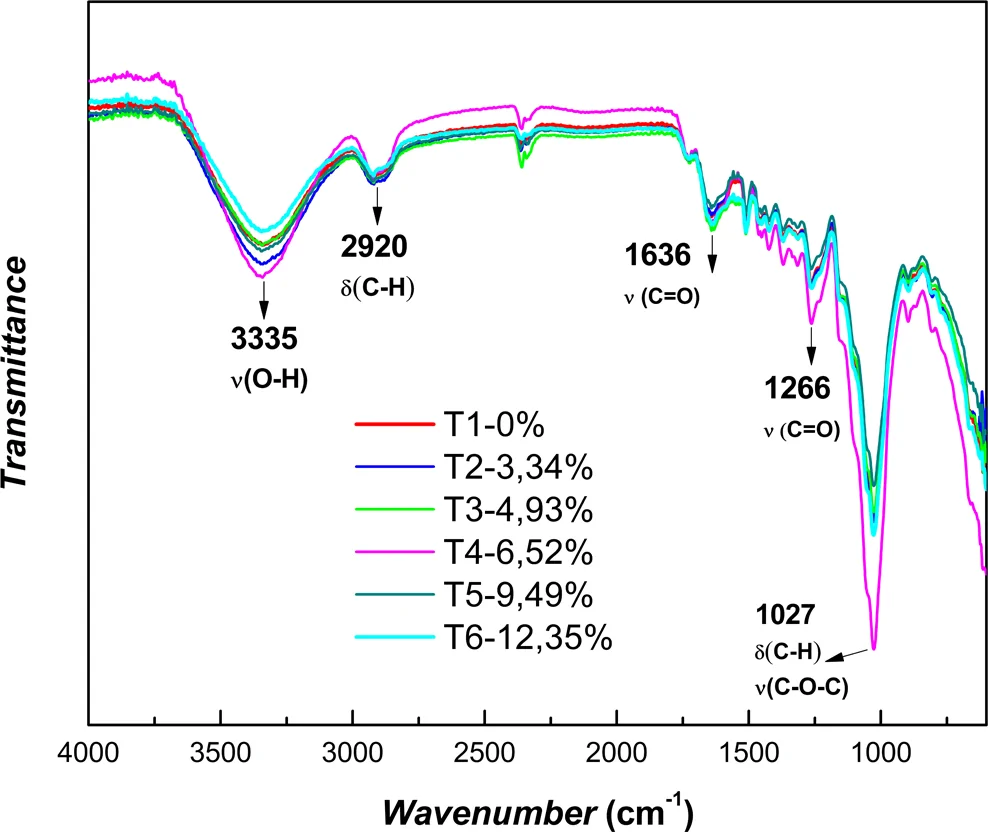
FTIR spectra of the particleboards with different filler proportions in the urea–formaldehyde (UF) adhesive.

The spectra observed in the particleboards showed a broad absorption band at approximately 3335 cm^–1^, attributed to O–H and N–H stretching vibrations.^79^ Signals at approximately 2920–2925 cm^–1^ are related to asymmetric C–H stretching vibrations in cellulose and lignin, predominantly in aromatic methyl groups, aromatic methoxyls, and methylene side chains.^80^ Bands between 1200 and 1600 cm^–1^ are characteristic of bond vibrations in the lignin molecule.^81^

The band at 1027 cm^–1^, associated with cellulose, also reflects interference from filler materials, corresponding to C–N stretching and N–C–N vibrations from methylene linkages.^82,83^ No distinct new absorption bands attributable exclusively to the glass wool–UF interaction were observed in the composite spectra. However, the absence of new bands alone cannot exclude interfacial interactions, particularly because the relatively low filler contents and the overlap between glass- and wood/UF-related bands may mask subtle spectral changes.

The characteristic glass-related bands observed at approximately 947 and 761 cm^–1^ were not reproduced as distinct, isolated bands in the composite spectra, which is consistent with their relatively low filler contents and the strong overlap with the absorption bands of the lignocellulosic/UF matrix. Nevertheless, differences in band shape and relative intensity were observed in the 900–1100 cm^–1^ region, as well as in the O–H/N–H stretching region around 3335 cm^–1^ and the C–H stretching region near 2920–2925 cm^–1^. These changes may reflect differences in phase contribution and/or interfacial interactions; however, the FTIR results do not provide sufficient evidence to assign them unequivocally to the formation of specific new chemical bonds.

## 4 Conclusions

The main findings indicate that glass wool acts predominantly as a physically dispersed, thermally stable inorganic phase, contributing to fire performance without inducing structural changes in the particleboards.

Most of the panels presented satisfactory performance in water-related properties, not exceeding the established normative limits.

The uniformity in density profiles indicates a consistent structure and effective process control across different treatments.

The treatment containing 3.34% glass wool was a positive highlight in the dimensional stability, and a higher content of glass wool enables improvement in surface resistance to fire.

The addition of glass wool did not induce detectable structural changes in the physical or chemical properties. However, the mineral filler contributed—albeit modestly—to the thermal stability of the boards, particularly during the final stages of thermal degradation.

Overall, this confirms the technical feasibility of incorporating glass wool waste into the urea–formaldehyde adhesive, with a potential reduction of the synthetic input. A quantitative environmental assessment, especially in the life cycle, is recommended for future studies.

## Data Availability

The authors declare that the data supporting the findings of this study are available within the paper. Any other raw data used in this study will be made available by the corresponding author upon reasonable request.
